# Supplementary figures and images for: Chromatin state architecture governs transcription factor accessibility across plant genomes
Source: PLoS Genet. 2026 Jan 22;22(1):e1012015. doi: 10.1371/journal.pgen.1012015 (PMC12867329; doi:10.1371/journal.pgen.1012015)

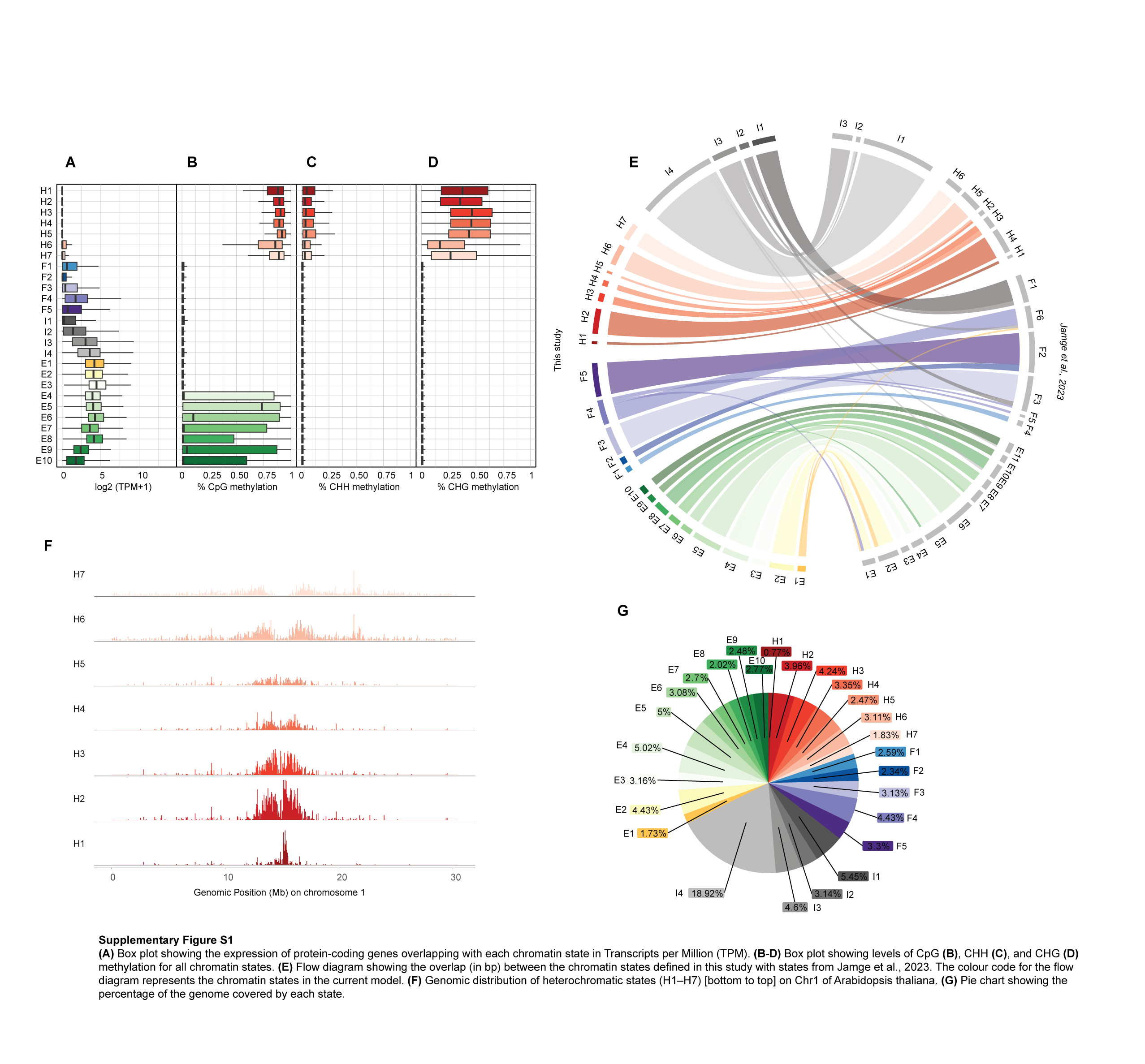

Supplement: S1 Fig — (A) Box plot showing the expression of protein-coding genes overlapping with each chromatin state in Transcripts per Million (TPM). (B-D) Box plot showing levels of CpG (B), CHH (C), and CHG (D) methylation for all chromatin states. (E) Flow diagram showing the overlap (in bp) between the chromatin states defined in this study with states from Jamge et al., 2023. The color code for the flow diagram represents the chromatin states in the current model. (F) Genomic distribution of heterochromatic states (H1–H7) from bottom to top on Chr1 of Arabidopsis thaliana. (G) Pie chart showing the percentage of the genome covered by each state. (TIF) [file pgen.1012015.s001.tif]

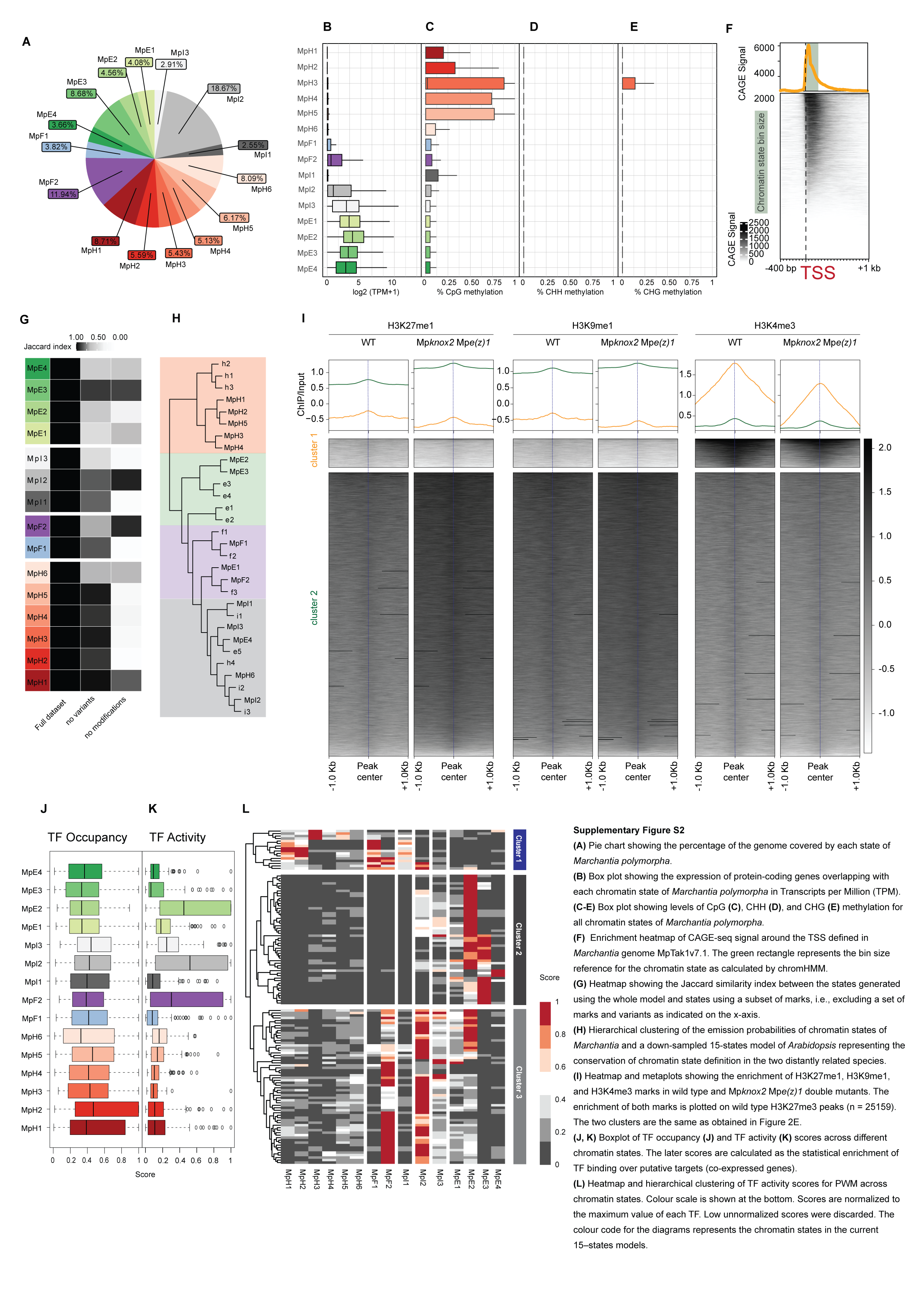

Supplement: S2 Fig — (A) Pie chart showing the percentage of the genome covered by each state of Marchantia polymorpha. (B) Box plot showing the expression of protein-coding genes overlapping with each chromatin state of Marchantia polymorpha in Transcripts per Million (TPM). (C-E) Box plot showing levels of CpG (C), CHH (D), and CHG (E) methylation for all chromatin states of Marchantia polymorpha. (F) Enrichment heatmap of CAGE-seq signal around the TSS defined in Marchantia genome MpTak1v7.1. The green rectangle represents the bin size reference for the chromatin state as calculated by chromHMM. (G) Heatmap showing the Jaccard similarity index between the states generated using the whole model and states using a subset of marks, i.e., excluding a set of marks and variants as indicated on the x-axis. (H) Hierarchical clustering of the emission probabilities of chromatin states of Marchantia and a down-sampled 15-states model of Arabidopsis representing the conservation of chromatin state definition in the two distantly related species. (I) Heatmap and metaplots showing the enrichment of H3K27me1, H3K9me1, and H3K4me3 marks in wild type and Mpknox2 Mpe(z)1 double mutants. The enrichment of both marks is plotted on wild type H3K27me3 peaks (n = 25159). The two clusters are the same as obtained in Fig 2E. (J, K) Boxplot of TF occupancy (J) and TF activity (K) scores across different chromatin states. The later scores are calculated as the statistical enrichment of TF binding over putative targets (co-expressed genes). (L) Heatmap and hierarchical clustering of TF activity scores for PWM across chromatin states. Colour scale is shown at the bottom. Scores are normalized to the maximum value of each TF. Low unnormalized scores were discarded. The colour code for the diagrams represents the chromatin states in the current 15–states models. (TIF) [file pgen.1012015.s002.tif]

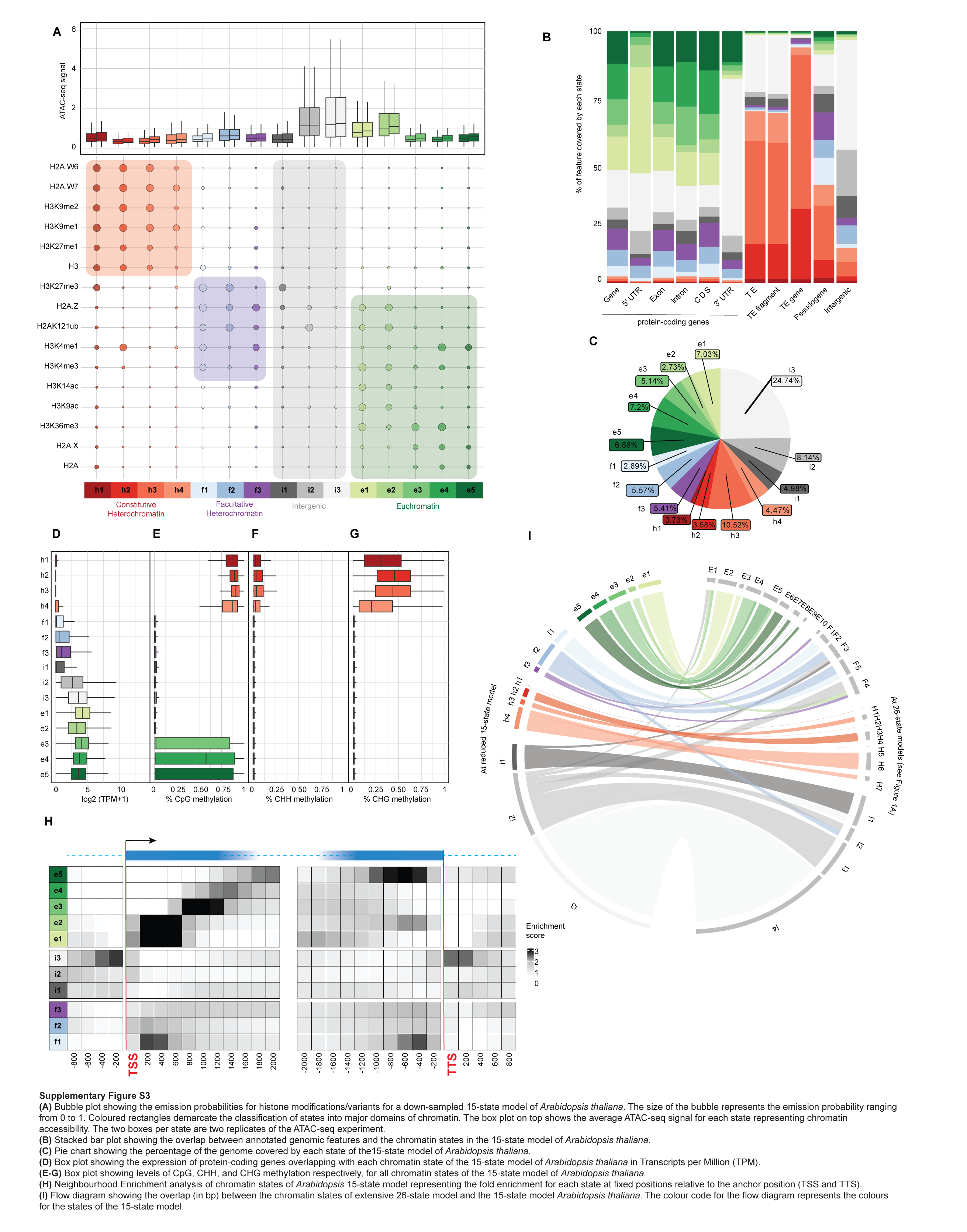

Supplement: S3 Fig — (A) Bubble plot showing the emission probabilities for histone modifications/variants for a down-sampled 15-state model of Arabidopsis thaliana. The size of the bubble represents the emission probability ranging from 0 to 1. Coloured rectangles demarcate the classification of states into major domains of chromatin. The box plot on top shows the average ATAC-seq signal for each state representing chromatin accessibility. The two boxes per state are two replicates of the ATAC-seq experiment. (B) Stacked bar plot showing the overlap between annotated genomic features and the chromatin states in the 15-state model of Arabidopsis thaliana. (C) Pie chart showing the percentage of the genome covered by each state of the 15-state model of Arabidopsis thaliana. (D) Box plot showing the expression of protein-coding genes overlapping with each chromatin state of the 15-state model of Arabidopsis thaliana in Transcripts per Million (TPM). (E-G) Box plot showing levels of CpG, CHH, and CHG methylation respectively, for all chromatin states of the 15-state model of Arabidopsis thaliana. (H) Neighbourhood Enrichment analysis of chromatin states of Arabidopsis 15-state model representing the fold enrichment for each state at fixed positions relative to the anchor position (TSS and TTS). (I) Flow diagram showing the overlap (in bp) between the chromatin states of extensive 26-state model and the 15-state model Arabidopsis thaliana. The colour code for the flow diagram represents the colours for the states of the 15-state model. (TIF) [file pgen.1012015.s003.tif]

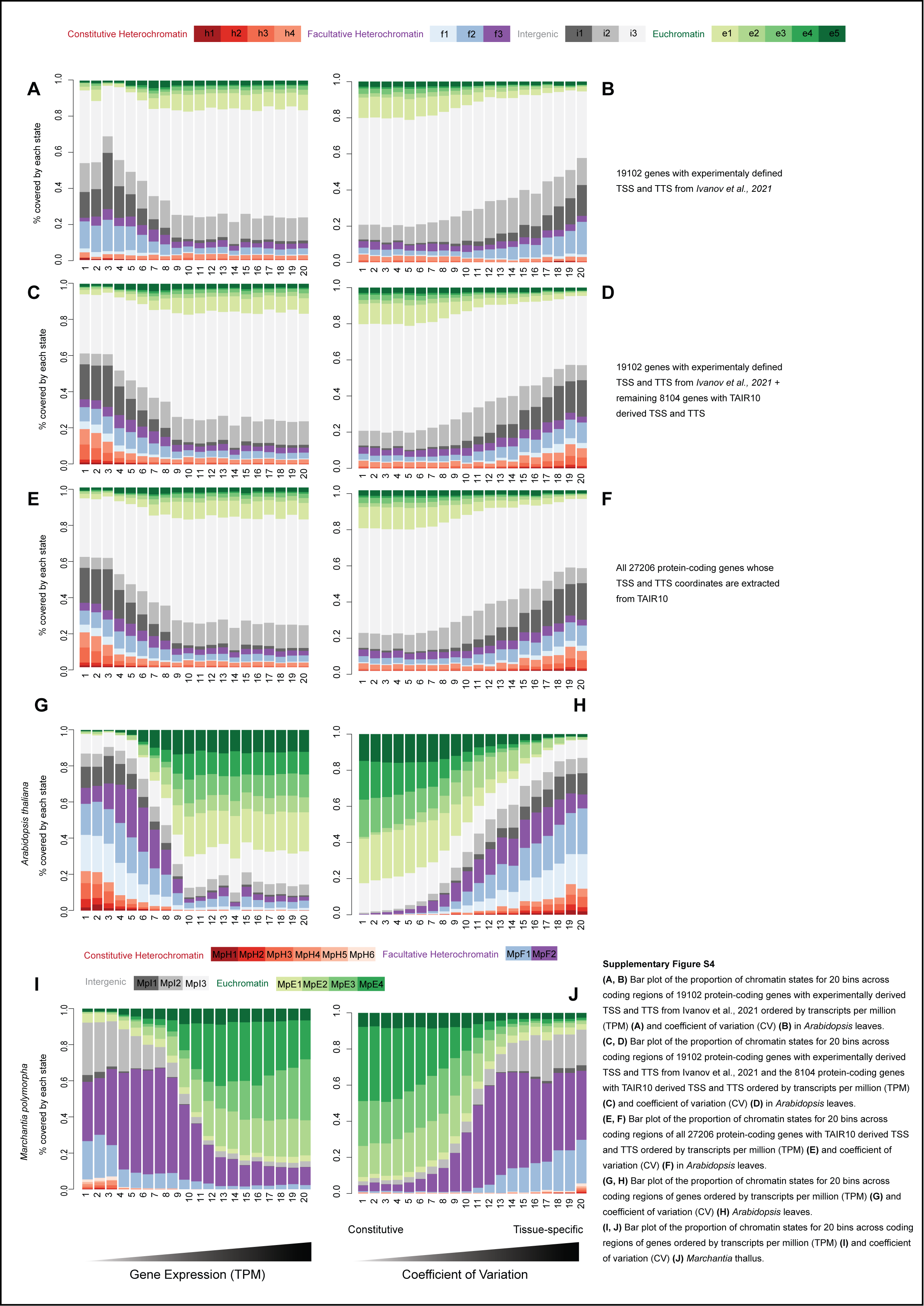

Supplement: S4 Fig — (A, B) Bar plot of the proportion of chromatin states for 20 bins across coding regions of 19102 protein-coding genes with experimentally derived TSS and TTS from Ivanov et al., 2021 ordered by transcripts per million (TPM) (A) and coefficient of variation (CV) (B) in Arabidopsis leaves. (C, D) Bar plot of the proportion of chromatin states for 20 bins across coding regions of 19102 protein-coding genes with experimentally derived TSS and TTS from Ivanov et al., 2021 and the 8104 protein-coding genes with TAIR10 derived TSS and TTS ordered by transcripts per million (TPM) (C) and coefficient of variation (CV) (D) in Arabidopsis leaves. (E, F) Bar plot of the proportion of chromatin states for 20 bins across coding regions of all 27206 protein-coding genes with TAIR10 derived TSS and TTS ordered by transcripts per million (TPM) (E) and coefficient of variation (CV) (F) in Arabidopsis leaves. (G, H) Bar plot of the proportion of chromatin states for 20 bins across coding regions of genes ordered by transcripts per million (TPM) (G) and coefficient of variation (CV) (H) Arabidopsis leaves. (I, J) Bar plot of the proportion of chromatin states for 20 bins across coding regions of genes ordered by transcripts per million (TPM) (I) and coefficient of variation (CV) (J) Marchantia thallus. (TIF) [file pgen.1012015.s004.tif]

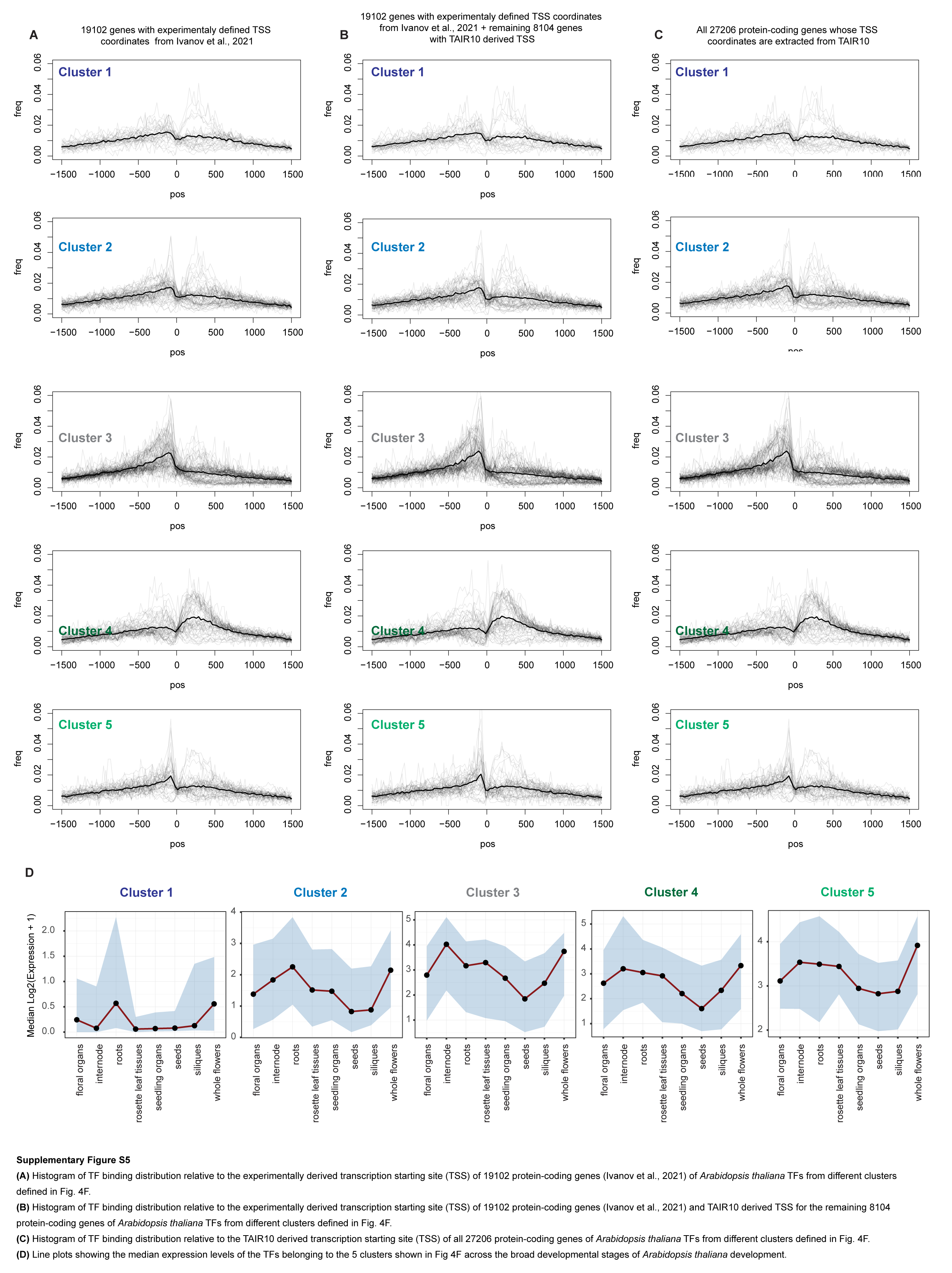

Supplement: S5 Fig — (A) Histogram of TF binding distribution relative to the experimentally derived transcription starting site (TSS) of 19102 protein-coding genes (Ivanov et al., 2021) of Arabidopsis thaliana TFs from different clusters defined in Fig 4F. (B) Histogram of TF binding distribution relative to the experimentally derived transcription starting site (TSS) of 19102 protein-coding genes (Ivanov et al., 2021) and TAIR10 derived TSS for the remaining 8104 protein-coding genes of Arabidopsis thaliana TFs from different clusters defined in Fig 4F. (C) Histogram of TF binding distribution relative to the TAIR10 derived transcription starting site (TSS) of all 27206 protein-coding genes of Arabidopsis thaliana TFs from different clusters defined in Fig 4F. (D) Line plots showing the median expression levels of the TFs belonging to the 5 clusters shown in Fig 4F across the broad developmental stages of Arabidopsis thaliana development. (TIF) [file pgen.1012015.s005.tif]

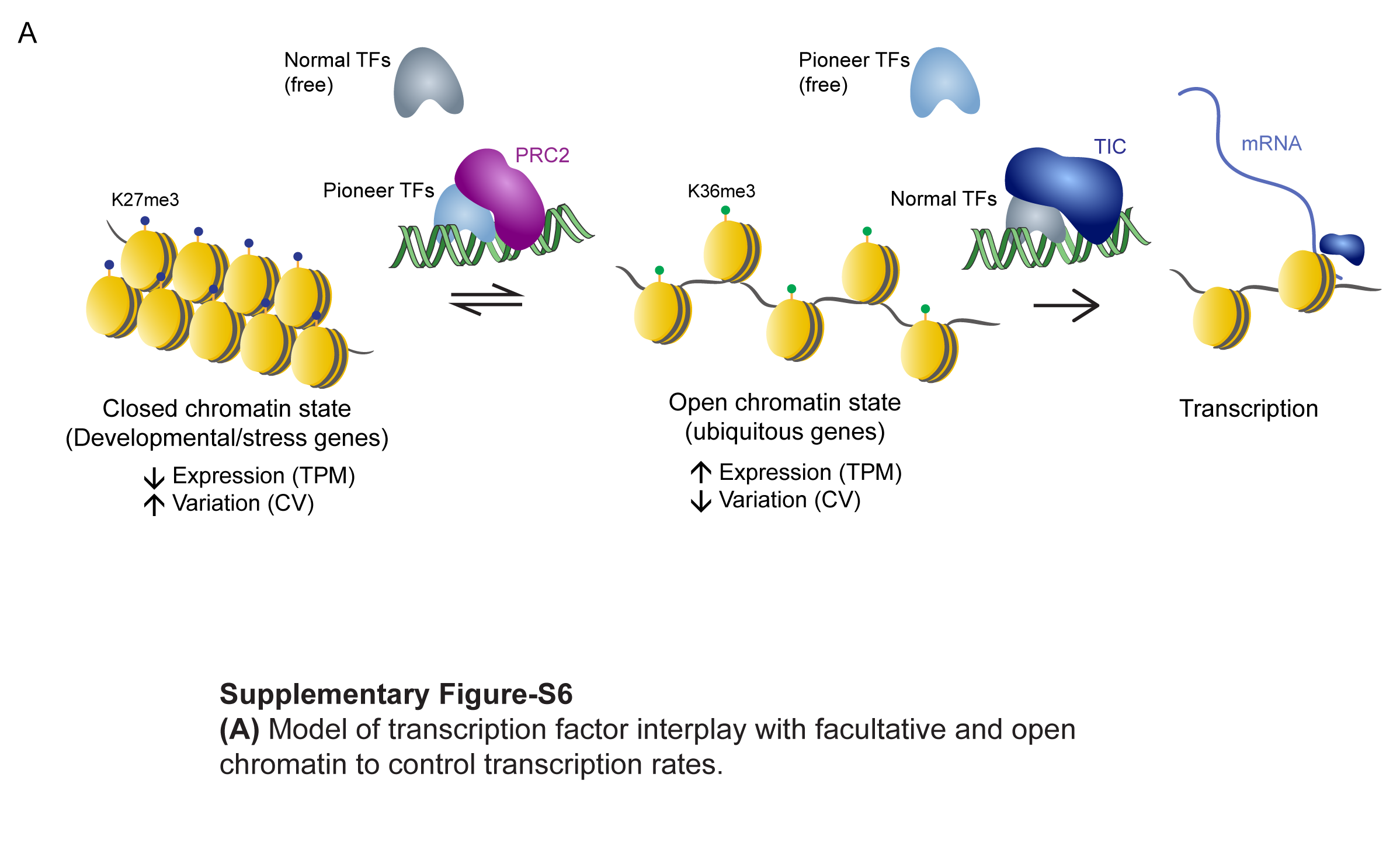

Supplement: S6 Fig — The histone icon was adapted from Bioicons: https://bioicons.com/icons/cc-by-4.0/Nucleic_acids/DBCLS/histone.svg. Acknowledgement: histone icon by DBCLS https://togotv.dbcls.jp/en/pics.html is licensed under CC-BY 4.0 Unported https://creativecommons.org/licenses/by/4.0/. (TIF) [file pgen.1012015.s006.tif]

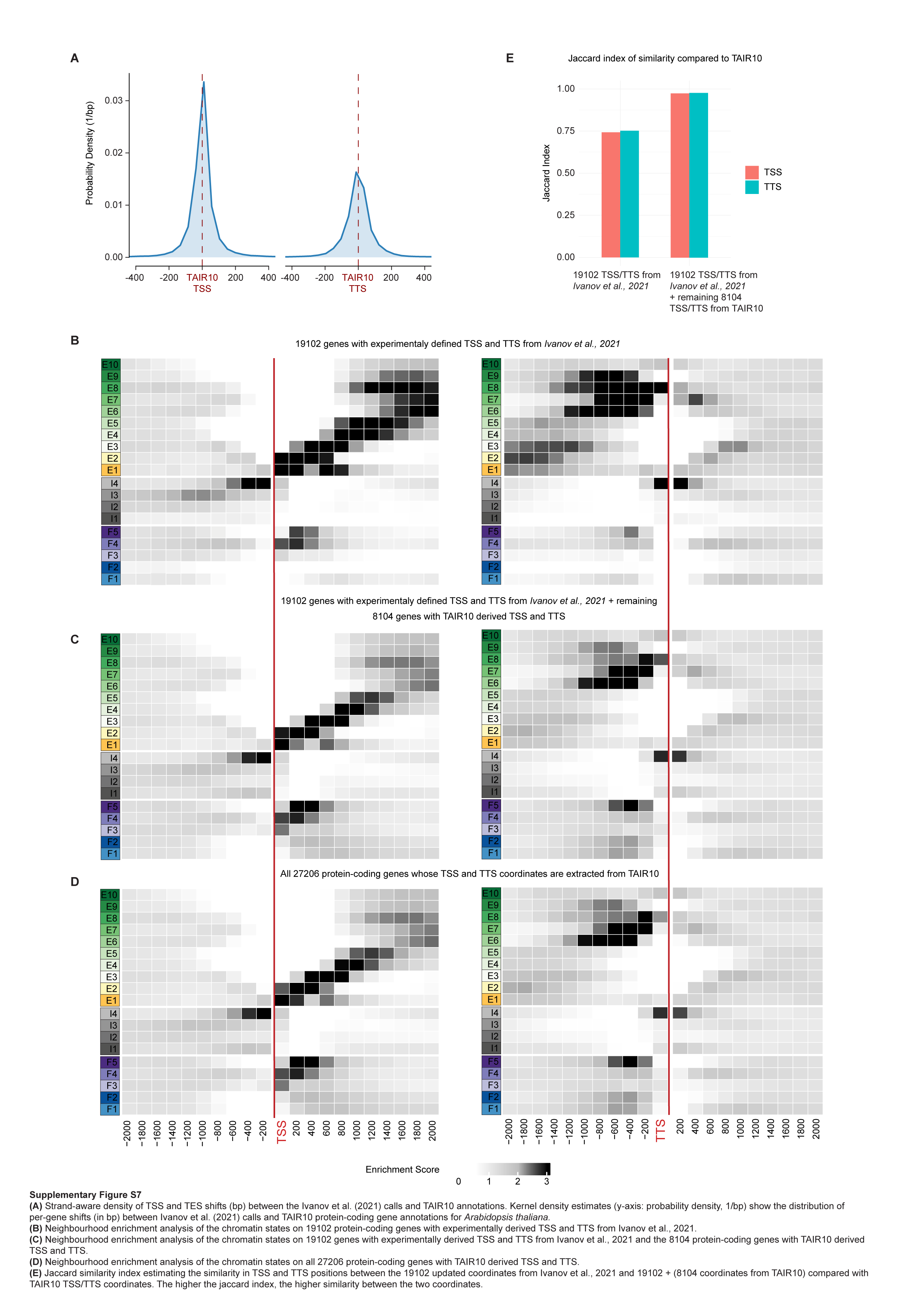

Supplement: S7 Fig — (A) Strand-aware density of TSS and TES shifts (bp) between the Ivanov et al. (2021) calls and TAIR10 annotations. Kernel density estimates (y-axis: probability density, 1/bp) show the distribution of per-gene shifts (in bp) between Ivanov et al. (2021) calls and TAIR10 protein-coding gene annotations for Arabidopsis thaliana. (B) Neighbourhood enrichment analysis of the chromatin states on 19102 protein-coding genes with experimentally derived TSS and TTS from Ivanov et al., 2021. (C) Neighbourhood enrichment analysis of the chromatin states on 19102 genes with experimentally derived TSS and TTS from Ivanov et al., 2021 and the 8104 protein-coding genes with TAIR10 derived TSS and TTS. (D) Neighbourhood enrichment analysis of the chromatin states on all 27206 protein-coding genes with TAIR10 derived TSS and TTS. (E) Jaccard similarity index estimating the similarity in TSS and TTS positions between the 19102 updated coordinates from Ivanov et al., 2021 and 19102 + (8104 coordinates from TAIR10) compared with TAIR10 TSS/TTS coordinates. The higher the jaccard index, the higher similarity between the two coordinates. (TIF) [file pgen.1012015.s007.tif]
