# Supplementary material for: Chromatin state architecture governs transcription factor accessibility across plant genomes
Source: PLoS Genet. 2026 Jan 22;22(1):e1012015. doi: 10.1371/journal.pgen.1012015 (PMC12867329; doi:10.1371/journal.pgen.1012015)
Supplement: Attachment — Submitted filename: 159915_1_rebuttal_3566340_t55rtg.pdf [file pgen.1012015.s016.pdf]

We thank the reviewers for their supportive comments and appreciate the suggestions provided. We have addressed each point in detail, and we provide below answers and how these modifications have improved our manuscript after revision.

Reviewer #1 (Evidence, reproducibility and clarity (Required))

The authors characterize chromatin states in the flowering plant Arabidopsis thaliana and the bryophyte Marchantia polymorpha. Here, they draw from ChIP-seq data that was previously published, and from data generated as part of this study, in particular for Marchantia H2.A variants (H2A.X.1, H2A.X.1, H2A.Z, H2A.M.2). The authors compute chromatin states, which enables a comparison over more than 450 million years of land plant evolution. While comparisons of plant chromatin to other species highlighted conservation as well as differences, this study targets a knowledge gap of evaluating chromatin conservation during land plant evolution. The authors investigate a connection between Transcription Factors binding sites and chromatin states. They propose a list of candidate pioneer factors associating with the +1 nucleosome.

Major comments

1. For the Association of chromatin states with expression, the authors use the TAIR10 annotation for extracting TSSs and promoter sequences. When investigated, a comparison of data resolving TSS with this annotation (or Araport11) shows a pretty poor overlap between the TSS based on Tair10/Araport11 and experimentally derived TSSs. This information was captured in Arabidopsis genome annotation files where the experimental TSS matches the genome annotation. What is the advantage of using an annotation with the inaccurate TSSs in TAIR10? It seems to confound the study.

Response: We thank Reviewer #1 for raising important point regarding the accuracy of TSS annotations used in our study. To address this concern, we used the experimentally derived TSS annotations for *Arabidopsis* from Ivanov et al. (2021). This dataset integrates multiple experimental approaches to provide high-confidence TSS positions. We mapped the experimentally determined TSS/TTS peaks from Ivanov et al. (2021) to their nearest TAIR10-annotated protein-coding genes, successfully updating the

TSS/TTS positions for 19,102 genes. When compared with the TSS from TAIR10 we observed that the TSS positions from Ivanov et al. (2021) showed most notable differences in a range of 200 bp on both sides from the TSS [See methods, Supplementary Figure S7A].

We used TSS/TTS positions derived from Ivanov et al. (2021) to evaluate the neighbourhood enrichment using three different TSS/TTS definitions: Set (1) 19,102 genes with experimentally updated TSS/TTS positions based on (Ivanov et al, 2021); Set (2) all 27,206 protein-coding genes, comprising the 19,102 genes with experimentally updated TSS/TTS and the remaining 8,104 genes with TAIR10-based TSS/TTS; and Set (3) all 27,206 protein-coding genes using only TAIR10-based TSS/TTS positions. The three sets showed very similar enrichment pattern of chromatin states on protein-coding genes [Supplementary Figure S7B–S7D]. To quantify the differences between the three matrices, we calculated the Jaccard similarity index comparing the set (1) and (2) with set (3). This comparison revealed that the matrices generated with TSS positions from Ivanov et al. (2021) are very similar with TAIR10 (Jaccard Index ~0.75) while the comparison between set (2) and TAIR10 showed a near identical match (Jaccard Index ~0.90) further explaining that the remaining differences between set (1) and TAIR10 are due to difference in total number of genes (19102 genes in set (1) and 27206 genes in TAIR10) and not due to different enrichment of states. These results demonstrate that our TSS annotation choice is sufficiently robust. Similarly, we did not observe a notable consequence of using either set of annotation for the preferential binding of TFs [Supplementary Figure S4A–F]. We think that the maximum divergence of 200bp in the position of TSS between TAIR10 and the other dataset would at the worst cause a shift by one nucleosome, which is the scale of resolution of our study. Hence it is logical that the imprecision of the TSS position in TAIR10 does not significantly affect our results. We have updated the manuscript to acknowledge both annotation sources and demonstrate the robustness of our findings across both datasets.

2. The TSS annotation in Marchantia polymorpha (Tak1 v7.1) may also match poorly to the experimentally derived TSS. I suggest that the authors generate data to detect TSS in their tissue of choice and compare the positions to the genome annotation they use (f.x. PMID: 38831668).

Response: We thank the reviewers for raising concerns about the accuracy of TSS annotations in *Marchantia polymorpha*. The current TSS annotations in MpTak1v7.1 are based on Iso-seq data (Montgomery et al, 2020), a long-read sequencing approach that accurately determines TSS positions, particularly for highly expressed genes. We further validated these annotations by examining CAGE-seq profiles, which revealed that most CAGE-seq peaks (Montgomery et al, 2020; Takahashi et al, 2012) aligned closely with the annotated TSS positions [Supplementary Figure S2F]. While this supports the general accuracy of current TSS annotations, refinements in TSS definitions may help reconcile the observed enrichment patterns. Our definition of TSS is further supported by the profile of enrichment in H2A.Z in *Marchantia* is similar to *Arabidopsis* and is so far the most universally conserved mark defining the +1 nucleosomes of expressed genes [Figure 2D].

It should also be noted that our chromatin state analysis uses 200 bp bins hence we cannot exclude the possibility that mapping of states on some genes may be offset by one bin (or ~1 nucleosome) in the upstream direction, an issue that future improvements in genome annotations may resolve.

Based on the current data, we cannot exclude the possibility of H3K36me3 and other elongation marks extending into promoter regions in state I3. However, given the inherent limitations in TSS positioning accuracy, future studies employing high-resolution TSS mapping will be essential to determine whether elongation marks are genuinely present at promoters or reflect the limitations of current TSS annotation.

3. I am not convinced that it is a wise choice to utilize fewer ChIP-seq data in Marchantia than Arabidopsis. Can the missing Marchantia ChIP-seq experiments not be performed and included to complete the comparison?

Response: We agree with Reviewer #1 that additional histone variant and modification datasets in *Marchantia* would be valuable for a more complete comparison. However, this would require generating new antibodies against *Marchantia* histone variants. Unfortunately, antibody generation is technically challenging with unpredictable success rates. We attempted to generate antibodies against each of the three H3 variants but were unable to obtain isoform-specific reagents. We also attempted to generate transgenic *Marchantia* lines expressing epitope-tagged H3 variants, but these transgenes were consistently silenced. Therefore, we proceeded with the currently

available datasets. The histone PTMs analyzed in *Marchantia* were selected based on their established roles in defining *Arabidopsis* chromatin states. Importantly, we demonstrate that reducing the number of histone marks in *Arabidopsis* slightly decreases the number of detected chromatin states but does not alter their fundamental characteristics [Supplementary Figure 1]. This analysis demonstrates that our comparison, while based on fewer marks in *Marchantia*, captures the core chromatin state architecture and represents the most comprehensive dataset currently available for this species. Generating additional datasets would require substantial technical development beyond the scope of this revision.

4. P. 26 onwards, the authors investigate different TF clusters and their association with chromatin states. They state "cluster 1 TFs primarily associated with the first nucleosome downstream of the TSS". However, if the gene is not really expressed in these "leave" tissues, then how can the authors be sure that the same TSS position would be used in "flower" tissue? It could be an artifact of a genome annotation file that misses flower-tissue TSS data. It is not an obvious conclusion to name these factors "pioneer TFs". Experiments testing this are missing as far as I can gather.

Response: We agree with Reviewer #1 that tissue-specific TSS usage could affect our interpretation. However, we note that cluster 1 contains several bona fide pioneer transcription factors previously demonstrated in *Arabidopsis*. We have revised the manuscript to use more cautious language, referring to these factors as "potential candidate pioneer TFs" throughout.

Minor comments

1. Can the authors add files (e.g. .bed) with their segmented chromatin states as part of their GEO submission? That could improve the impact and make the findings more accessible.

Response: This data is now added and publicly accessible as additional data files.

2. Can the authors rule out issues with the Marchantia annotation, for example missing read-through transcription or alternative isoforms, that would essentially have the effect that the genomic segmentation they use contains elongating upstream transcripts in from of promoter TSS? This could be an alternative explanation for the enrichment of H2AUb/H3K36me3 just upstream of the TSSs as they describe on p.21. If it can´t be ruled, the limitations from genome annotations, and examples offering improvements could be highlighted in the discussion. This may also be supported by the long persistence of E4 after the TTS p.23.

Response: We thank Reviewer #1 for this insightful suggestion regarding potential annotation issues. Our CAGE-seq analysis [Supplementary Figure S3H, discussed above] does not show evidence of widespread read-through transcription for the genes examined. However, long-read transcriptome data in *Marchantia* reveal alternative isoforms and transcript variants for some genes, which could potentially affect chromatin state assignments in specific cases. Comprehensive re-annotation of the *Marchantia* genome is beyond the scope of this revision. We have added this caveat to both the Results and Discussion sections, noting: "The enrichment of H3K36me3 and H2Aub upstream of TSS in some genes may reflect incomplete transcript annotations, including unannotated read-through transcription or alternative isoforms. Similarly, the extended persistence of state E4 downstream of the TTS may also reflect annotation limitations, such as unannotated 3' UTR extensions or downstream transcriptional activity. Future improvements in genome annotation will help resolve this question." We agree this warrants further investigation.

3. P.23 - This further suggests that in Marchantia, the orientation of genes defines distinct chromatin environment in their vicinity, through mechanisms yet to be uncovered. Does this correlate with the distance of the closest (annotated) transcript pairs?

Response: We thank Reviewer #1 for this insightful suggestion. We examined whether intergenic distance correlates with the orientation-dependent chromatin patterns observed in *Marchantia*. Unlike *Arabidopsis*, where genes and TEs are largely segregated, the *Marchantia* genome shows extensive interspersion of genes and transposable elements, which could potentially influence local chromatin environments.

However, our analysis did not reveal systematic patterns linking intergenic distance or TE proximity to the observed chromatin state differences. Nevertheless, we cannot exclude the possibility that read-through transcription or inaccuracies in TTS annotation may contribute to these patterns. We have acknowledged this limitation in the revised manuscript and note that future improvements in transcript annotation will help clarify the mechanisms underlying orientation-dependent chromatin organization in *Marchantia*.

"The marks H3K4me1 and H3K36me3 reflecting transcriptional elongation and confined to the gene bodies in Arabidopsis, extend beyond the TTS in Marchantia, suggesting that signals for transcriptional termination differ between flowering plants and bryophytes. However, we cannot exclude that this pattern may result from a combination of technical limitations including missing transcripts in the current MpTak1v7.1 genome annotation (e.g., lncRNAs) or potential annotation errors (e.g., incorrect gene ends or read-through transcripts)."

4. The E1 state highlighted on p.24 and in Fig.3A/d is not annotated in Fig.3A/D. It is also not clear in the legends which number it is.

Response: We thank Reviewer#1 for pointing this error out. This is now corrected.

5. P.30 - The marks H3K4me1 and H3K36me3 reflecting transcriptional elongation and confined to the gene bodies in Arabidopsis, extend beyond the TTS in Marchantia, suggesting that signals for transcriptional termination differ between flowering plants and bryophytes. There are multiple alternative explanations. Likely a combination of missing transcripts in their genome annotation (e.g. lncRNAs), annotation errors (e.g. wrong ends) and the segmentation of these regions (e.g. the transcripts are closer than in Arabidopsis). The discussion could extended significantly to address these issues and include the efforts to improve the genome annotations.

Response: We thank the reviewer for the suggestions which are now included in the discussion.

Referee cross-commenting

Reviewer #2 raises fair and valuable questions.

Reviewer #1 (Significance (Required)):

Significance

The authors corroborate prior chromatin state analyses in Arabidopsis and provide a chromatin state analysis for Marchantia. These data represent a resource that will be used and appreciated by the plant and ChromEvoDevo communities. The quality of the analyses are high and the description is transparent. I am not aware of a similar study comparing bryophytes and a land plant, so this study addresses a gap in knowledge.

General assessment:

The quality of the manuscript is high. The analyses are described well, and in sufficient detail to be understood. The effort going into documentation is high, I rate the study as reproducible. The linked github deposition looks good. The data generated as part of this study is available in the linked GEO deposition. An experimental design of 2 biological repeats is used, which is OK, but the lower limit. The GEO-deposited .bw files should be of interest to the ChromEvoDevo community, and researchers interested in Marchantia epigenetics and gene expression. The manuscript is written clearly and to the point. The figures condense a lot of data and match the text. The figures are rather complex and not easily accessible to someone browsing through a journal issue. However, that is fine for these types of papers. The manuscript is strong on data analysis. Other approaches, for example mutants to validate their hypothesis, are not utilized. The calculation of chromatin states offers a way to condense complex information into simpler terms. Nevertheless, it re-organizes information that largely existed before. To me, the biggest value of this study appears to be to regard it as a resource that calculated the chromatin states in a comparable fashion between organisms.

Advance: The manuscript provides several advances. It provides new ChIP-seq data for Marchantia, it generates a chromatin state map for Marchantia, it compares Chromatin

state maps between distant evolutionary time, and it generates a new hypothesis regarding pioneer TFs in plants. Some of the points described in the article hold true for even larger evolutionary distances, for example comparing plants to yeast and metazoans. The manuscript fills a knowledge gap and has offers a comparison via the computation of comparable chromatin states.

Audience: The audience will be colleagues interested in chromatin and epigenetics, the Marchantia and plant communities as well as researchers interested in EvoDevo of chromatin organization. Even though the study uses plant models, it is highly relevant for non-plant models.

Reviewer #2 (Evidence, reproducibility and clarity (Required))

The manuscript by Shukla et al described the "chromatin states" in the bryophyte Marchantia polymorpha and compared it with that in Arabidopsis thaliana. They described the generally common features of chromatin states between these evolutionally distant plant species, but they also find some differences. The authors also studied the connection between chromatin states and TF bindings, mostly in Arabidopsis due to the scarcity of the TF binding data in Marchantia. Their analyses lead to interesting finding that specific transcription families tend to associate with specific chromatin state, which tend to associate with specific genomic regions such as promoter, TSS, gene body, and fucultative heterochromatin. Overall, the authors provide novel piece of information regarding the evolutional conservation of chromatin states and the relationship between chromatin states and TFs.

Major comments

1. In the end of the abstract they state "The association with the +1 nucleosome defines a list of candidate pioneer factors we know little about in plants", which is one of their major points. This is based on the results Fig4F and 4G, described in P27 L16-17. Question is, is cluster 1 TFs really associated with the +1 nucleosome? From Fig. 1C, +1 nucleosome is characterized mostly by E1 state and also by E2, F3, F4. However, from Fig. 4F, cluster 1 TFs are not associated with E1/E2 and association is not particularly strong for F3/F4. Indeed association with E1/E2 is

much conspicuous for cluster 4 TFs. Therefore, authors should reconsider this point and consider rephrasing or showing further results of analyses.

Response: We thank Reviewer #2 for highlighting this important point, which reflects a lack of clarity in our presentation. States E1/E2 indeed mark the +1 nucleosome position in actively expressed genes. However, cluster 1 TFs preferentially bind to genes that are occupied by constitutive heterochromatin states in the tissue analyzed (2-week-old seedlings) [Supplementary Figure S5D]. These target genes are not expressed in this tissue. Consequently, the +1 nucleosome position at these genes is marked by heterochromatic states rather than E1/E2. We have revised the text to clarify that cluster 1 TF binding at the +1 nucleosome position of repressed genes suggests potential pioneer activity—the ability to access chromatin in a repressed state. This interpretation is consistent with the known properties of validated pioneer factors.

2. P17 last line to P18, they state "The facultative heterochromatin states were primarily associated with the intergenic states I1 to I3, based on their enrichment in H3K27me3 and H2AK121ub, low accessibility, and low gene expression". I'm not sure about this statement. How can they say "primarily associated" from the data they cite? As far as the PTMs and variants patterns, I1 to I3 and facultative heterochromatin look different. The authors should explain more or rephrase.

Response: we thank reviewer #2 for pointing out the lack of clarity in reporting this point. We have rephrased this section to:

"Contrasting with the intergenic state I4, the other intergenic states I1, I2 and I3 were enriched in H3K27me3 and H2AK121ub, and showed lower gene expression [Figure 1A and Supplementary Figure S1A]. These features suggest that the intergenic states I1, I2, and I3 are likely related to facultative heterochromatin, together occupying ca 30% of the genome [Supplementary Figure S1G]."

3. P20 L15, the authors state "Contrary to Arabidopsis, the promoters of Marchantia defined by the region just upstream of the TSS showed enrichment of H2AUb and the elongation mark H3K36me3, along with other euchromatic marks. " I have a concern that the TSS annotation could be inaccurate in Marchantia compared to

more rigorously tested annotation of Arabidopsis thaliana, so that the relationship between TSS and histone PTMs could be different between species. The authors should make sure this is not the case.

Response: We thank both the reviewers for raising concerns about the accuracy of TSS annotations in *Marchantia polymorpha*. The current TSS annotations in MpTak1v7.1 are based on Iso-seq data (Montgomery et al, 2020), a long-read sequencing approach that accurately determines TSS positions, particularly for highly expressed genes. We further validated these annotations by examining CAGE-seq profiles, which revealed that most CAGE-seq peaks (Montgomery et al, 2020; Takahashi et al, 2012) aligned closely with the annotated TSS positions [Supplementary Figure S2F]. While this supports the general accuracy of current TSS annotations, refinements in TSS definitions may help reconcile the observed enrichment patterns.

It should also be noted that our chromatin state analysis uses 200 bp bins hence we cannot exclude the possibility that mapping of states on some genes may be offset by one bin (or ~1 nucleosome) in the upstream direction, an issue that future improvements in genome annotations may resolve.

Interestingly, the profile of enrichment in H2A.Z is similar to *Arabidopsis* and is so far the most universally conserved mark defining the +1 nucleosomes of expressed genes [Figure 2D].

Based on the current data, we cannot exclude the possibility of H3K36me3 and other elongation marks extending into promoter regions in state I3. However, given the inherent limitations in TSS positioning accuracy, future studies employing high-resolution TSS mapping will be essential to determine whether elongation marks are genuinely present at promoters or reflect the limitations of current TSS annotation.

4. P21 last line to P22, they analyzed only H3K27me3 and H2Aub in the mutants of E(z) (Fig. 2E) and states that "we analyzed chromatin landscape in the Marchantia...". Is analyzing two histone marks enough to say "chromatin landscape"?

Response: We thank Reviewer#2 for pointing out this oversight, which is now corrected as follows:

"To further compare the impact of PRC2 on histone modifications that define in a major manner the chromatin states , we analysed enrichment of H3K27me3, H2Aub, H3K4me3, H3K9me1, and H3K27me1 in the Marchantia Mpknox2 Mpe(z)1 double mutant, which exhibited reduced PRC2 activity (Shu et al, 2019; Hisanaga et al, 2023b) [Figure 2E, Supplementary Figure S2I]."

5. In addition, they state "These findings suggest a strong independence of the two Polycomb repressive pathways in Marchantia. " However, they did not analyzed the effect of loss of PRC1 on H3K27me3; the opposite way. Actually, in Arabidopsis loss of PRC1 causes loss of H2Aub AND H3K27me3 (Zhou et al (2017) Genome Biol: DOI 10.1186/s13059-017-1197-z).

Response: We thank Reviewer#2 for pointing out this oversight, which is now corrected as follows:

"Conversely, the loss of PRC1 affected the deposition of both H3K27me3 and H2Aub (Zhou et al, 2017) suggesting a partial independence of the two Polycomb repressive pathways in Marchantia."

6. Related to the above comments, they states "To further compare the regulation by PRC2 in both species,". However, they did not describe the knowledge about regulation by PRC2 in Arabidopsis. They should consider describing.

Response: This section was rewritten with additional references were cited.

7. P25 L14: "With this method to estimate TF activity, the scores of TF occupancy and activity converged. To look at different patterns of chromatin preferences among TFs, we kept ChIP-seq and DAP-seq data for ~300 TFs in Arabidopsis (after filtering out TFs with low scores of occupancy and activity)." This part is a little hard to follow. Perhaps better to explain in more detail.

Response: We extended the description of this section to allow space for more detail on how the analysis was performed and the logic behind it.

8. In discussion section P30 L19-21: "This could be due to open chromatin, which is associated with highly expressed genes and permissive for TF binding, generating highly occupied target regions (HOT) with redundant or passive activity (19)." This part needs further explanation; espetially for the latter part, It's not clar what the authors claim.

Response: We thank Reviewer#2 for raising this point. We clarified this point to make the discussion more precise with the literature. We do not claim that all binding in the HOT regions is redundant or passive. In fact, our results support that there is a correlation with co-expression. Yet, some binding events might be product of the open chromatin configuration and then redundant or passive.

Minor comments

We thank Reviewer#2 for pointing out these mistakes and they have been corrected.

1. P17 L21: H2bUb should be H2Bub.

Response: We thank Reviewer#1 for pointing this error out. This is now corrected.

2. Legend of Fig. 4D: later should be latter.

Response: We thank Reviewer#1 for pointing this error out. This is now corrected.

3. Legend of Fig. 4G and H: "clusters defined in figure-H" should be "defined in Fig. 4F"?

Response: We thank Reviewer#1 for pointing this error out. This is now corrected.

Referee cross-commenting

Reviewer #1 raises thorough and important points that should be addressed before the manuscript is published. Particularly about the comparison of chromatin states between Arabidopsis and Marchantia, as this paper will make foundation for further research in

the future and serve as a resource for community, the authors should thoroughly look into the points raised by reviewer #1 including annotation of transcriptional units.

Reviewer #2 (Significance (Required)):

Strength and limitation:

Strength of this paper is the insights into chromatin-based transcriptional regulation by defining chromatin states using combination of many epigenome data and compare it with TF biding data. Limitation is lack of experimental support for their interesting claims by perturbing histone PTMs, for example. Also, a limitation is that comparing only two species can tell subjective "similar" or "different" between species.

Advance comparing past literature:

One clear advance is studying chromatin states in a plant other than Arabidopsis thaliana. Another one is revealing that TFs can be classified into a number of groups according to the relationships with chromatin-based transcription regulation. However, experimental tests for these are awaited.

Audience:

Epigenetics, chromatin, and transcription researchers, plant biologists interested in transcriptional regulation.

My expertise:

Epigenome, genetics, histone PTMs, plants
